# Cortical Excitability and Connectivity in Patients With Brain Tumors

**DOI:** 10.3389/fneur.2021.673836

**Published:** 2021-08-26

**Authors:** Vincenzo Rizzo, Carmen Terranova, Giovanni Raffa, Salvatore Massimiliano Cardali, Filippo Flavio Angileri, Giuseppina Marzano, Maria Catena Quattropani, Antonino Germanò, Paolo Girlanda, Angelo Quartarone

**Affiliations:** ^1^Department of Clinical and Experimental Medicine, University of Messina, Messina, Italy; ^2^Division of Neurosurgery, BIOMORF Department, University of Messina, Messina, Italy; ^3^Department of Biomedical Science and Morphological and Functional Images, University of Messina, Messina, Italy

**Keywords:** cortical excitability, brain tumors, transcranial magnetic stimulation, motor function, recovery

## Abstract

**Background:** Brain tumors can cause different changes in excitation and inhibition at the neuronal network level. These changes can be generated from mechanical and cellular alterations, often manifesting clinically as seizures.

**Objective/Hypothesis:** The effects of brain tumors on cortical excitability (CE) have not yet been well-evaluated. The aim of the current study was to further investigate cortical–cortical and cortical–spinal excitability in patients with brain tumors using a more extensive transcranial magnetic stimulation protocol.

**Methods:** We evaluated CE on 12 consecutive patients with lesions within or close to the precentral gyrus, as well as in the subcortical white matter motor pathways. We assessed resting and active motor threshold, short-latency intracortical inhibition (SICI), intracortical facilitation (ICF), short-latency afferent inhibition (SAI), long-latency afferent inhibition, cortical silent period, and interhemispheric inhibition.

**Results:** CE was reduced in patients with brain tumors than in healthy controls. In addition, SICI, ICF, and SAI were lower in the affected hemisphere compared to the unaffected and healthy controls.

**Conclusions:** CE is abnormal in hemispheres affected by brain tumors. Further studies are needed to determine if CE is related with motor impairment.

## Introduction

The “maximal safe resection” represents the goal standard of the modern surgical treatment of brain tumors located in eloquent areas. Various techniques supply important anatomical and functional information regarding the brain functional organization. Different neuroimaging and neurophysiological techniques can be used to plan a surgical strategy to preserve functional networks and to increase the maximal safe resection. Navigated transcranial magnetic stimulation (nTMS) is a helpful tool for preoperative cortical mapping and planning before surgery of brain tumors located in eloquent areas (1–5). Brain tumors like brain trauma, hematoma, and focal cerebral ischemia can cause brain parenchyma compression that can produce changes in excitation and inhibition, even in the absence of histologically significant cell injury, often manifesting clinically as seizures. The precise mechanism producing seizures after cortical compression remains elusive (6). Recent studies used preoperative nTMS as a predictor of motor outcome in patients with brain tumors. Rosenstock et al. showed that an abnormal interhemispheric resting motor threshold (RMT) ratio was related to a higher risk for poor postoperative outcome in the 1st week, but not in the following 3 months (7). This proposed stratification model, based on functional–anatomical and neurophysiological measures, could allow quantification of the functional impairment or recovery potential. Several parameters of cortical excitability have been studied in patients with traumatic brain injury (8) and stroke (9). In a recent paper, Neville et al. (10) described an increase in motor threshold (MT) that was paralleled by an alteration in short-latency intracortical inhibition (SICI) and intracortical facilitation (ICF) (10). However, the authors did not check other important parameters of cortical excitability such as short-latency afferent inhibition (SAI), long-latency afferent inhibition (LAI), cortical silent period (CSP), recruitment curve (RC), and interhemispheric inhibition (IHI). The aim of the current study was to further investigate cortical–cortical and cortical–spinal excitability in patients with brain tumors using a more extensive TMS protocol.

## Materials and Methods

The present study was conducted in accordance with the ethics committee of the University of Messina and the Declaration of Helsinki. Informed consent was obtained from every patient.

### Patient Population

The cortical excitability measurements by transcranial magnetic stimulation (TMS) were carried out on 12 consecutive patients with lesions involving the primary motor cortex (M1) and corticospinal tract (CST). Not all 12 patients participated in all cortical excitability measurements (see below). Table 1 reports all nosographic data and neurological status of patients. Exclusion criteria for brain stimulation were the same as for magnetic resonance imaging (MRI). Patients were examined for handedness, motor impairment, medical history, and use of medication (see Table 1). The WHO classification was used for tumor histology (11).

**Table 1:** Summary of patients' epidemiological data.

	**Age, sex**	**Handedness**	**Tumor location**	**Neurological examination**	**Histology**	**AEDs**
#1, DM	51, F	R	MC I, right	Moderate left upper limb weakness	Oligodendroglioma IDH-mutant 1p/19q-codeleted	LEV
#2, BA	38, M	R	MC I left	No deficit	Oligodendroglioma IDH-mutant 1p/19q-codeleted	//
#3, FT	69, F	R	Fronto-temporo-insular, left	No deficit	Glioblastoma IDH-wildtype	//
#4, CS	35, M	R	Fronto-temporo-insular, right	No deficit	Diffuse astrocytoma IDH-wildtype	LEV
#5, CG	46, M	R	Fronto-insular, left	No deficit	Glioblastoma IDH-mutant	//
#6, CT	70, F	R	Fronto-opercular, left	No deficit	Gliobastoma IDH-wildtype	LEV
#7, VA	67, M	R	Fronto-temporal, right	No deficit	Gliobastoma IDH-wildtype	//
#8, CG	46, M	L	Fronto-temporal, right	No deficit	Diffuse astrocytoma IDH-wildtype	//
#9, PN	53, F	R	Fronto-temporal, right	No deficit	Oligodendroglioma NOS	//
#10, MR	60, F	R	Temporo-parietal, left	No deficit	Gliobastoma IDH-wildtype	LEV
#11, MW	36,M	L	Fronto-temporo-insular, right	No deficit	Diffuse astrocytoma IDH-wildtype	LEV
#12, BAG	55,M	R	Temporo-parietal, right	No deficit	Gliobastoma IDH-wildtype	LEV

*Nosographic data and neurological status for all 12 consecutive patients with lesions within or close to the precentral gyrus*.

*MC, motor cortex; LEV, levetiracetam*.

### Measures of Cortical Excitability

Patients and controls were seated in a “comfortable reclining chair and surface EMG was recorded from the right or left first dorsal interosseus (FDI) muscle using disposable disc electrodes with a belly-tendon montage. EMG was filtered by Neurolog System supplied by Digitimer with a time constant of 3 ms, and a high pass filter set a 3 kHz.” Single or paired pulses were given to the right or left M1 using a standard figure-of-eight coil connected with a single (for single-pulse TMS) or two (for paired-pulse TMS) high-power Magstim 200 stimulators. “Signals were collected via a CED 1401 laboratory interface (Cambridge Electronic Design, Cambridge, UK) and fed to a personal computer for offline analysis” (12).

### Threshold Measurements

In 10 patients, we evaluated RMT and active MT (AMT). “RMT was defined as the minimum intensity that evoked a peak-to-peak motor evoked potential (MEP) of 50 μV in at least 5 out of 10 consecutive trials in the relaxed FDI muscle. AMT was defined as the minimum intensity that elicited a reproducible MEP of at least 200 μV in the tonically contracting FDI muscle in at least 5 out of 10 consecutive trials” (13).

### Recruitment Curve

In 12 patients, we evaluated input–output RC. “Motor evoked potentials (MEPs) input–output recruitment curve was performed at stimulus intensities ranging from 100 to 150% RMT (in steps of 10%). Fifteen peak-to-peak MEP at each stimulation intensities were averaged” (12).

### Intracortical Paired-Pulse Excitability

In 10 patients, we studied SICI and ICF. SICI and ICF were determined according to the paired-pulse method described by Kujirai et al. (14). The intensity of the conditioning stimulus was set at 80% of AMT, while the test stimulus was adjusted to elicit MEPs with amplitudes of 0.5–1.0 mV at baseline (115–125% of RMT in healthy subjects, and ~140–150% of the RMT in patients with brain tumors). SICI and ICF were assessed at ISIs of 2 and 12 ms, respectively. The mean amplitude of the conditioned MEP was expressed as percentage of the amplitude of the unconditioned MEP. This characterized the strength of SICI and ICF.

### Cortical Silent Period

In eight patients, we evaluated CSP. CSP was measured during slight tonic contraction of the right or left FDI muscle at ~10–15% of maximum force level measurements. The intensity of the test stimulus was 130% of resting MT. The duration of the CSP was measured in each trial (15).

### Sensorimotor Intracortical Inhibition

In nine patients, we studied SAI and LAI, which were studied using the conditioning test protocol described by Tokimura et al. (16). The median nerve was stimulated through bipolar electrodes at the wrist (cathode proximal). The intensity was set just approximately three times the perceptual threshold. The intensity of the transcranial test stimulus was adjusted to evoke a muscle response in relaxed abductor pollicis brevis (APB) with a peak-to-peak amplitude of ~0.5–1 mV (115–125% of RMT in healthy subjects, and ~140–150% of RMT in patients with brain tumors). SAI and LAI were probed at ISIs of 20, 25, and 200 ms, respectively. The relative change in MEP amplitude induced by the peripheral stimulus was taken as a measure of SAI and LAI.

### Interhemispheric Inhibition

“A conditioning-test protocol as described by Ferbert et al. (17) was used to evaluate IHI of the right or left M1. IHI was studied in 8 patients. A conditioning stimulus was applied to the left or right M1, and the test stimulus was applied to the homologous right or left M1. We set the intensity of the first (conditioning) stimulus to obtain an inhibition of the test MEP to about 50% at an ISI of 10 ms. The second (test) stimulus was set at an intensity that, when given alone, would evoke an EMG response of 0.5–1 mV peak-to-peak amplitude (115–125% of RMT in healthy subjects, ~140–150% of the RMT in patients with brain tumors). IHI was tested at three conditioning-test intervals (8, 9, 10 ms)” (18).

### Statistical Analysis

Factorial ANOVA was computed to show differences in RMT; SAI 20 and 25 ms; LAI; SICI; ICF; IHI at 8, 9, and 10 ms; and CSP between the affected hemisphere, unaffected hemisphere, and controls. MEP RCs were evaluated in separate repeated-measures ANOVA in the different sets of subjects. We performed a two-way repeated-measures ANOVA with intensity (six levels: 100, 110, 120, 130,140, 150% of MT) as within-subject factor, and group (three levels: affected hemisphere, unaffected hemisphere, and controls) as between-subjects factor. If appropriate, *post hoc t*-tests were performed. *Post hoc* Fisher's PLSD analysis was executed for RC. Significance was set at *p* < 0.05. Data are given as mean ± standard error of the mean.

## Results

No participants reported any adverse effects during or after the study.

### Motor Threshold

**Figure 1:**
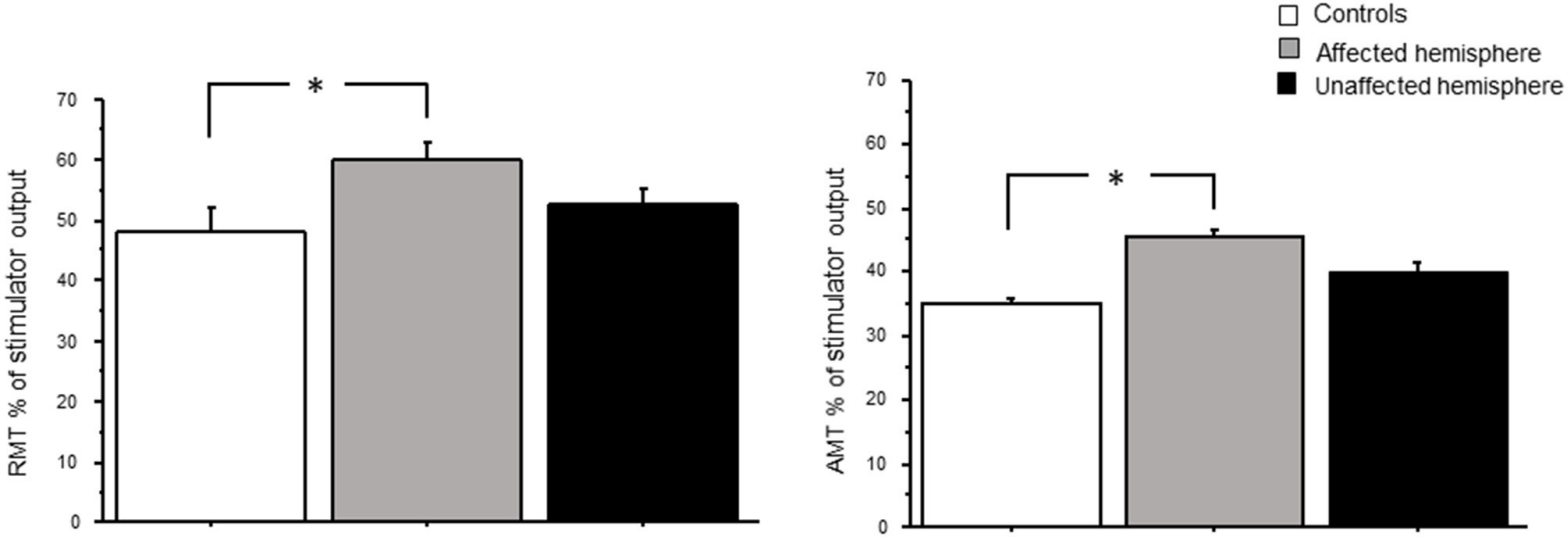
RMT and AMT levels in controls and patients with brain tumors (affected and unaffected hemispheres). RMT and AMT are expressed as % of max output. *p < 0.05.

RMT and AMT were significantly higher in patients than in controls [RMT: *F*_(2,26)_ = 4.03, *p* = 0.029; power = 0.66; AMT: *F*_(2,26)_ = 4.1, *p* = 0.028, power = 0.65] (Figure 1). *Post hoc t-*tests revealed relative change only between controls and the affected hemisphere of patients [for RMT: *t*_(1,9)_ = 2.6, *p* = 0.017; for AMT: *t*_(1,9)_ = 2.2, *p* = 0.03].

### Recruitment Curve

**Figure 2:**
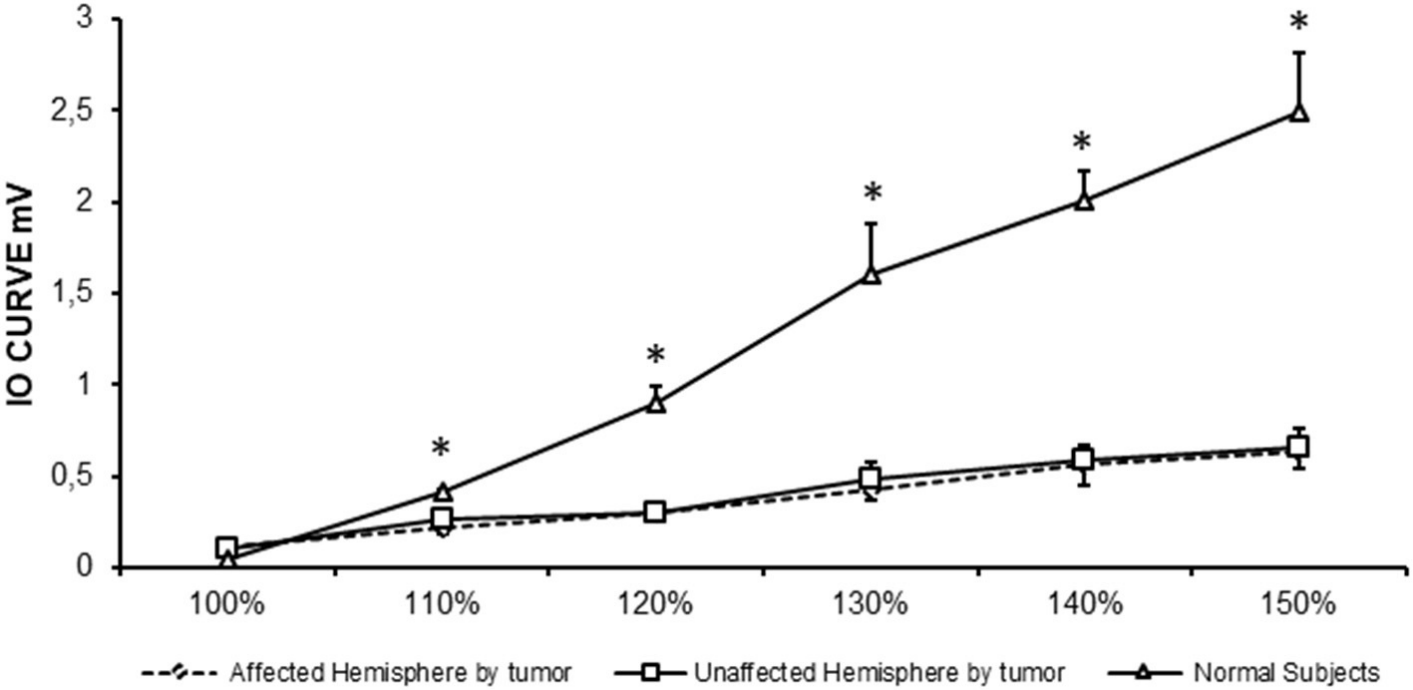
RC in controls and patients with brain tumors (affected and unaffected hemispheres). Stimulus intensities ranging from 100 to 150% RMT (in steps of 10%). *p < 0.05.

MEP amplitudes increased with increasing stimulus intensity in controls and patients. However, MEP RC was significantly less steep in patients in both hemispheres compared to controls (Figure 2). Indeed, repeated ANOVA indicated a significant effect for intensity [*F*_(2,29)_ = 50.9, *p* < 0.0001; power = 1.0] with a significant interaction between intensity and groups [*F*_(2,29)_ = 18.780, *p* < 0.0001; power = 1.0]. *Post hoc* Fisher's PLSD analysis showed that MEP amplitudes were significantly higher in control subjects compared to the affected (*p* < 0.001) and unaffected hemispheres of patients (*p* < 0.001). On the contrary, there were no differences between the RCs of both affected and unaffected hemispheres (*p* = 0.9) of patients.

### Intracortical Paired-Pulse Excitability

**Figure 3:**
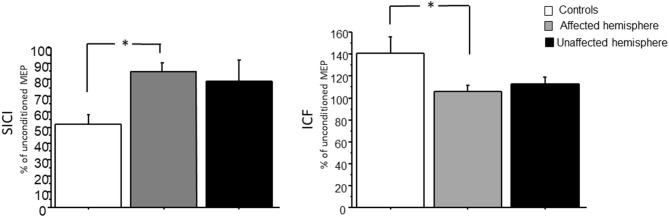
SICI and ICF in controls and patients with brain tumors (affected and unaffected hemispheres). Mean amplitude of the conditioned MEP was expressed as percentage of the amplitude of the unconditioned MEP. The relative change in MEP amplitude induced by the conditioning stimulus characterized the strength of SICI and ICF. *p < 0.05.

Paired-pulse stimulation consistently produced SICI at an ISI of 2 ms and ICF at an interval of 12 ms in controls, but not so well in patients. The data showed a lower degree of inhibition and facilitation in brain tumor patients (Figure 3). ANOVA showed a main effect between patients (affected and unaffected hemispheres) and controls for ICI [*F*_(2,27)_ = 3.87, *p* = 0.03; power = 0.65] and ICF [*F*_(2,27)_ = 3.58, *p* = 0.042; power = 0.58]. *Post hoc t*-tests revealed relative change only between controls and affected hemisphere of patients for ICI [*t*_(1,9)_ = 4.10, *p* = 0.0006] and ICF [*t*_(1,9)_ = 2.13, *p* = 0.046], but none between controls and unaffected hemisphere of patients [ICI: *t*_(1,9)_ = 1.8, *p* = 0.08; ICF: *t*_(1,9)_ = 1.7, *p* = 0.1].

### Sensorimotor Intracortical Inhibition

**Figure 4:**
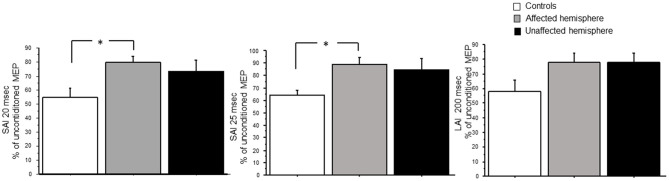
SAI at 20 and 25 ms, LAI at 200 ms in controls and patients with brain tumors (affected and unaffected hemispheres). The relative change in MEP amplitude induced by the peripheral stimulus was taken as a measure of SAI and LAI. *p < 0.05.

ANOVA showed a selective reduction in SAI (20 and 25 ms) but not in LAI (200 ms) (see Figure 4). For SAI, there was a prominent main effect for ISI at 25 ms [*F*_(2,29)_ = 5.33; *p* = 0.01; power = 0.8] and 20 ms [*F*_(2,29)_ = 4.29; *p* = 0.02; power = 0.7]. *Post hoc t-*tests revealed significant change only between controls and affected hemisphere of patients for SAI at 25 ms [*t*_(1,9)_ = 3.6, *p* = 0.0015] and 20 ms [*t*_(1,9)_ = 2.7, *p* = 0.011]. For LAI, ANOVA demonstrated no main effect [*F*_(2,29)_ = 2.7; *p* = 0.08].

### Cortical Silent Period

**Figure 5:**
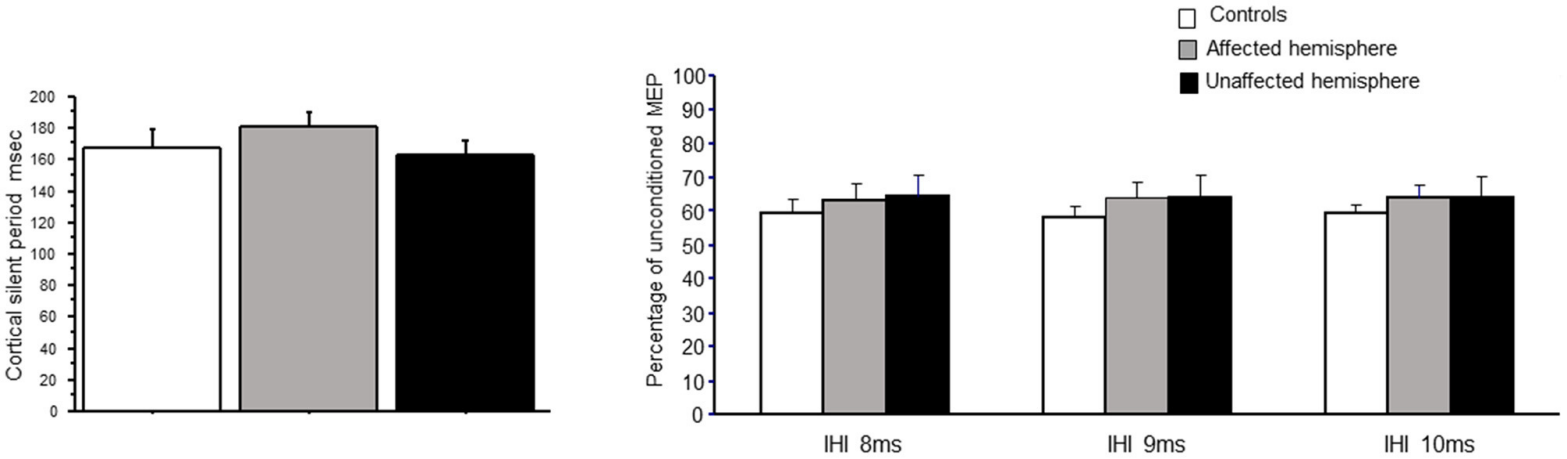
CSP and IHI at 7, 8, and 9 ms in controls and patients with brain tumors (affected and unaffected hemispheres).

The duration of CSP did not differ between patients (affected and unaffected hemispheres) and controls [*F*_(2,21)_ = 0.75; *p* = 0.48] (see Figure 5).

### Interhemispheric Inhibition

Figure 5 shows also the time course of IHI in patients (affected and unaffected hemispheres) and controls. Repeated-measures ANOVA did not reveal a significant interaction between the two main factors of ISI and population for no interval [8 ms: *F*_(2,23)_ = 3.17; *p* = 0.07; 9 ms: *F*_(2,23)_ = 2.34; *p* = 0.11; 10 ms: *F*_(2,23)_ = 2.82; *p* = 0.08].

## Discussion

Our data yielded three main findings. First, affected and unaffected hemisphere excitability in patients with brain tumors was reduced compared to healthy controls. Second, SICI, ICF, and SAI were lower in the affected hemisphere. Third, IHI, LAI, and CSP showed no differences between patients and healthy controls. These findings indicate that the effects of brain tumors on cortical excitability are mostly localized to the affected hemisphere.

### Effects on Corticospinal Excitability

MT is an indicator of cortical excitability reflecting membrane excitability (19). In our study, RMT and AMT were significantly higher in patients, especially in the affected hemisphere, than in healthy controls. This result may be due to a reduced density and number of corticospinal neurons in relation to motor impairment. However, in our study, only one patient had a moderate left upper limb weakness. In addition, higher MT may predict a poor motor outcome in patients with brain tumors (20). Picht et al. speculated that patients initially without hemiparesis but with high RMT were at a higher risk in the long term of a decline in motor function (20). Rosenstock et al. studied abnormal RMT interhemispheric ratio was related to a higher risk for poor postoperative outcome in the 1st week, but not in the following 3 months (7). Similar to previous findings, Neville et al. reported an increase in MT in patients with brain tumors (10). Furthermore, in stroke patients, Swayne et al. (21) demonstrated that corticospinal excitability of the affected hemisphere, measured as AMT and RMT, increased in the acute phase, but this increment became weaker at 3 months and it continued for 6 months (chronic phase of stroke). The authors concluded that, in the chronic phase of stroke, the motor function could be dependent on the reorganization of alternative cortical networks (21). Moreover, our data show that MEP RC was significantly less steep in patients in both hemispheres compared to controls. RC illustrates a graded profile of cortical spinal tract (CST) function, providing a more global measure of cortical excitability than MT (22). Alterations in the slope of the RC can predict more substantial CST damage, motor impairment, and poor recovery in patients with brain injury (23). RCs are widely used in stroke because they are believed to reflect CST gain and output from the primary motor cortex (24). In our study, MEP RC was significantly less steep in patients in both hemispheres compared to controls. Experimental data in animal model suggest that glioma cells release high amounts of glutamate resulting in excitotoxicity and tumor invasion (25, 26). Therefore, it is likely that excitotoxicity at a chronic stage may result in a significant loss of motor neurons within primary motor area and a loss of CSTs as indexed by the increase in RMT and AMT and by the reduced slope of RC. The excitotoxicity promoted by infiltrating glioma cells may also in turn affect fast-spiking GABA_A_ interneurons, and a reduced GABA availability may create a vicious circle where increased pyramidal neurons firing amplify glutamate excitotoxicity (27), producing neuronal cell death (see below).

### Effects on Cortical Excitability

We showed that SICI, ICF, and SAI are reduced in the affected hemisphere compared to the unaffected and healthy controls. On the other hand, there were no significant differences of LAI and CSP between patients and healthy controls. SICI is mediated by GABA_A_ receptors, while CSP is a marker for the excitability of long-lasting (presumably GABA_B_) intracortical interneurons. Conversely, ICF is mediated by glutamate and is associated with excitatory cortical circuits (28–30). In a case report, only the lack of inhibition, assessed by SICI, had been demonstrated in two patients with focal motor seizures caused by a circumscribed glioblastoma or metastasis (31). The results of our study are in agreement with similar findings of Neville et al., who reported an alteration of SICI and ICF in the affected hemisphere (10). The novel finding of a reduced SICI and ICF in the affected hemisphere could be caused by the simultaneous selective reduction in GABAergic_A_ inhibition and glutamatergic excitation because of either reduced excitability or loss of inhibitory/excitatory neurons or changes in GABAergic/glutamatergic receptor function. The presence of a disruption of GABA_A_ mechanisms in the affected hemisphere could explain the high percentage of seizures (50%) in our population. On the other hand, this disinhibition could also be an adaptive plastic mechanism recruited by the affected hemisphere to counteract the reduced overall excitability caused by tumor-related brain edema and swelling (see above). These findings parallel evidences in stroke patients where SICI is reduced in the affected hemisphere in the first 6 months with normal CSP and may contribute to cortical reorganization and recovery (32).

This reduced GABA_A_ intracortical inhibition is in line with several findings coming from the bench side. The largest class (40–50%) of GABAergic interneurons is represented by fast-spiking, parvalbumin-positive cells, which are present in all layers and form synapses on the soma and proximal dendrites of pyramidal cells (33). Several pieces of evidence in animal models have demonstrated a loss of fast-spiking interneurons, and more in general a reduced firing rate in the peritumoral glioma area (34). This dysfunction of fast-spiking GABAa interneurons is critically involved in tumor-associated epileptic seizures (34).

Interestingly, it has been demonstrated that the selective optogenetic stimulation of parvalbumin-positive GABA_A_ interneurons induces a significant reduction in glioma cell proliferation (27). Human glioblastoma cells may express functional GABA_A_ receptors, and that endogenous GABA release may attenuate tumor proliferation (35). On the other hand, pyramidal cell stimulation enhances cell proliferation in tumor mass (36). It is likely that fast-spiking GABA_A_ interneuron vulnerability to tumor-induced excitotoxicity may trigger a vicious circle where reduced GABA availability may increase pyramidal neuron firing, producing an enhanced tumor growth. Future studies in humans are needed to better understand the relationship of SICI with tumor proliferation.

Another important finding was a selective reduction in SAI (20 and 25 ms) but not in LAI (200 ms) in the affected hemisphere. LAI and SAI are mediated through different sensorimotor circuits. SAI is controlled by muscarinic neurotransmission (37). LAI is significantly understudied compared to SAI, and the neural circuitries underlying these phenomena are unclear (38). SAI has been used to assess and predict functional recovery following ischemic stroke, where larger SAI reductions correlate with improved long-term recovery 6 months following injury (39). The presence of a reduced SAI in the affected hemisphere of patients with brain tumor needs to be better investigated in future longitudinal studies, especially in low-grade glioma, to see if SAI reduction is heralding a good recovery after surgical operation.

### Effects on IHI

Our study demonstrates no differences in IHI between patients and controls. The normalcy of IHI confirms previous evidence suggesting that a physiological interplay between the two primary motor cortices is required to maintain a good motor function in patients with brain tumor (40). In a very elegant study, brain connectivity was measured in patients with glioma using task-free functional MRI to probe motor networks. Patients with motor weakness showed reduced interhemispheric and left primary motor cortex and the right premotor area connectivity compared to healthy controls. Conversely, in patients without motor deficit, motor performance assessed on the grooved pegboard was not related to interhemispheric connectivity, which was unchanged but correlated with ipsilateral connectivity between the premotor area and supplementary motor area (40).

The absence of motor impairment in our cohort of patients could be explained by the normalcy of transcallosal connection evaluated using the IHI. Indeed, Otten et al. showed that an integrity of transcallosal pathway between the two motor areas is needed to maintain a normal motor function (40). Since TMS is a non-invasive technique, future longitudinal studies are warranted to explore the role of IHI to predict motor outcome in patients with brain tumor.

## Conclusions

This study investigates cortical–cortical and cortical–spinal excitability in patients with brain tumors using a more extensive TMS protocol. Different measurements of cortical excitability are abnormal in brain hemispheres affected by tumors, but further studies are needed to determine their relationship to motor impairment and subsequent motor recovery. Finally, it will be important to explore the correlation between brain tumor molecular features and the impairment of cortical excitability in a lager sample population.

## Data Availability Statement

The raw data supporting the conclusions of this article will be made available by the authors, without undue reservation.

## Ethics Statement

The studies involving human participants were reviewed and approved by AOU G. Martino. The patients/participants provided their written informed consent to participate in this study.

## Author Contributions

CT, GR, GM, SC, and FA contributed to the acquisition, analysis and interpretation of data, and writing of the first draft. VR and AQ were responsible for the conception and design, data acquisition, analysis and interpretation as well as the review, and critique of the manuscript. MQ, PG, and AG contributed to interpretation of data and the review and critique of the final manuscript. All authors contributed to the article and approved the submitted version.

## Conflict of Interest

The authors declare that the research was conducted in the absence of any commercial or financial relationships that could be construed as a potential conflict of interest.

## Publisher's Note

All claims expressed in this article are solely those of the authors and do not necessarily represent those of their affiliated organizations, or those of the publisher, the editors and the reviewers. Any product that may be evaluated in this article, or claim that may be made by its manufacturer, is not guaranteed or endorsed by the publisher.
